# Refractive index measurements of solid deuterium–tritium

**DOI:** 10.1038/s41598-022-06298-1

**Published:** 2022-02-15

**Authors:** Keisuke Iwano, Jiaqi Zhang, Akifumi Iwamoto, Yuki Iwasa, Keisuke Shigemori, Masanori Hara, Yuji Hatano, Takayoshi Norimatsu, Kohei Yamanoi

**Affiliations:** 1 Institute of Laser Engineering, Osaka University, 2-6 Yamadaoka, Suita, Osaka 565-0871 Japan; 2 National Institute for Fusion Science, 322-6 Oroshi, Toki, Gifu 509-5292 Japan; 3 National Metrology Institute of Japan (NMIJ), National Institute of Advanced Industrial Science and Technology (AIST), 1-1-1 Central 3, Umezono, Tsukuba, Ibaraki 305-8563 Japan; 4 Faculty of Science, Academic Assembly, University of Toyama, 3190 Gofuku, Toyama, 930-8555 Japan

**Keywords:** Atomic and molecular physics, Optical physics, Nuclear fuel, Nuclear fusion and fission, Nuclear waste

## Abstract

Physical properties of tritium (T) and deuterium (D) have been of great interest as a fuel for nuclear fusion. However, several kinds of the physical properties in a cryogenic environment have not been reported. Optical properties in liquid and solid phases are indispensable for the quality control of the DT fuel. We study the dependence of the refractive index of solid DT on temperature. A dedicated cryogenic system has been developed and forms a transparent solid DT in a prism cell. Refractive index measurements based on Snell’s law were conducted. The refractive indexes of solid DT are from 1.1618 ± 0.0002 to 1.1628 ± 0.0002 in the temperature range of 19.40 K to 17.89 K.

## Introduction

Hydrogen (H), deuterium (D), and tritium (T) should have notable isotope effects because of their mass difference. The comparison of physical properties between hydrogen and deuterium has been reported^1^. However, in terms of tritium, few physical properties have been studied experimentally. In particular, measurements in a solid state with high density must be conducted in a high radioactive environment. Most of the data come from empirical estimations. For nuclear fusion, solid deuterium–tritium (DT) is a candidate as the fuel. In magnetic fusion, the DT solid fuel is supplied for experiments in the Joint European Tokamak (JET)^2^. ITER will start DT operation in 2035^3^ and the DEMO reactors applying the DT fuel have been designed^4^. In inertial fusion, the experiments with a DT fuel are conducted at the National Ignition Facility (NIF)^5^. Koyo-fast and LIFT, which are prototype and experimental reactor respectively, have been designed on the basis of the DT fuel^6,7^. The high precise sphericality on the DT fuel is required for the inertial fusion. The in-situ methods to confirm the fuel quality and quantity are required because the fuel must be supplied continuously. The method of applying the refractive index is one of the attractive candidates to assay the D/T ratio of DT fuel. Previously, the voidless solid hydrogen and uniform layering technique have been experimentally established, and the hydrogen refractive index with the temperature dependence has been measured^8,9^. It is known that the refractive index of HD depends on the composite ratio of H and D^10^. The refractive index of DT should also depend on the DT ratio. Therefore, we can know the DT ratio by measuring the refractive index. The refractive indexes of D_2_ and DT (D:T = 1:1), *n,* are calculated using the equation by Briggs et al.^10^

1 $$\left(n-1\right)=\left[\left(3.15\pm 0.12\right)\cdot {10}^{-6}\right]\cdot \rho $$

where *ρ* is the density in moles per cubic meter of D_2_ or DT. However, the values are empirically estimated and have the large error bar of ± 0.006. The precise refractive index values are required within ± 0.001 accuracy of solid DT^11,12^. In this study, we experimentally measured the precise refractive index of DT and its temperature dependence.

## Methods

### Preparation of D–T mixture

**Figure 1:**
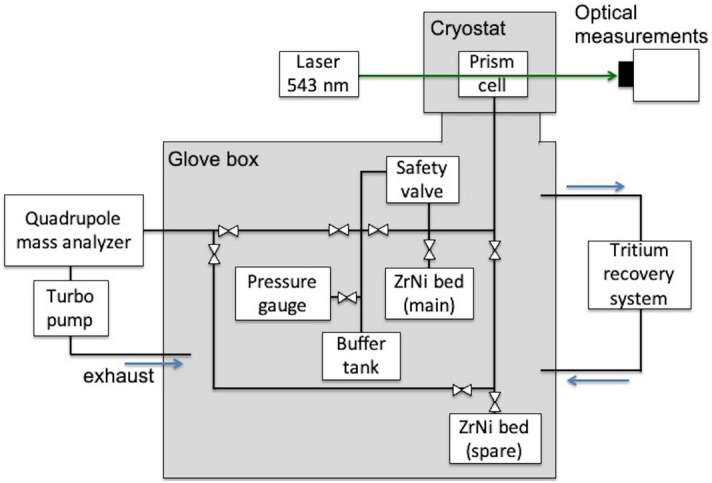
Configuration of the gaseous DT supply system.

Figure 1 shows the schematic diagram of the tritium handling system. The system is composed of the optical measurement, tritium storage, and gas control parts. The tritium handling system is enclosed in the glove box which is connected with the tritium recovery system (TRS). For the preparation of gaseous DT, the glass bottle of gaseous T_2_ from American Radiolabeled Chemicals Inc. (St. Louis, MO. USA) was connected to the handling system, and gaseous T_2_ was introduced into the handling system. The amount of gaseous T_2_ was determined by the pressure–volume-temperature method. It was found to be 2.68 × 10^6^ Pa cc and the activity was estimated to be 2.54 TBq. Subsequently, gaseous T_2_ was completely absorbed to a ZrNi alloy bed as metal tritide^13,14^. Hydrogen isotope gases with the equilibrium composition at high temperature are released from the metal hydride. Gaseous D_2_ of 2.88 × 10^6^ Pa cc was also loaded into the ZrNi bed to obtain the almost equimolar volume of D and T. The composition of D-T was confirmed by the quadrupole mass spectrum analyzer (Qulee CGM-052, ULVAC, Kanagawa, Japan) via an orifice to reduce the pressure from 100 Pa to below 0.01 Pa. The flow speed calibration of hydrogen isotopes through the orifice was carried out using H_2_ and D_2_ in advance. The flow velocity ratio of D/H is √1/2 corresponding to the square root of the mass ratio at pressures below 500 Pa. The flow velocity ratio of T/H was assumed to be the square root of the mass ratio of √1/3. This isotope effect was caused by the difference in the velocity of hydrogen isotopes through the orifice. The sensitivity factors of the mass spectrum analyzer are D_2_/H_2_ = 0.99, DT/H_2_ = 0.96, and T_2_/H_2_ = 0.88^15^. Consequently, the ratio of H:D:T in the gaseous DT was 1:54:45. The 1% of H came from the vacuum line made of stainless steel because H was used in preliminary experiments with the same system. The uncertainty of the ratio is less than 1% which was estimated from the sensitivity of mass spectrum analyzer. The isotopologue composition was estimated to be around D_2_:DT:T_2_ = 3:5:2. The solidification and refractive index measurements were carried out within 5 days after gaseous DT was absorbed in ZrNi.

### Experiment with cryogenic and DT supply system

**Figure 2:**
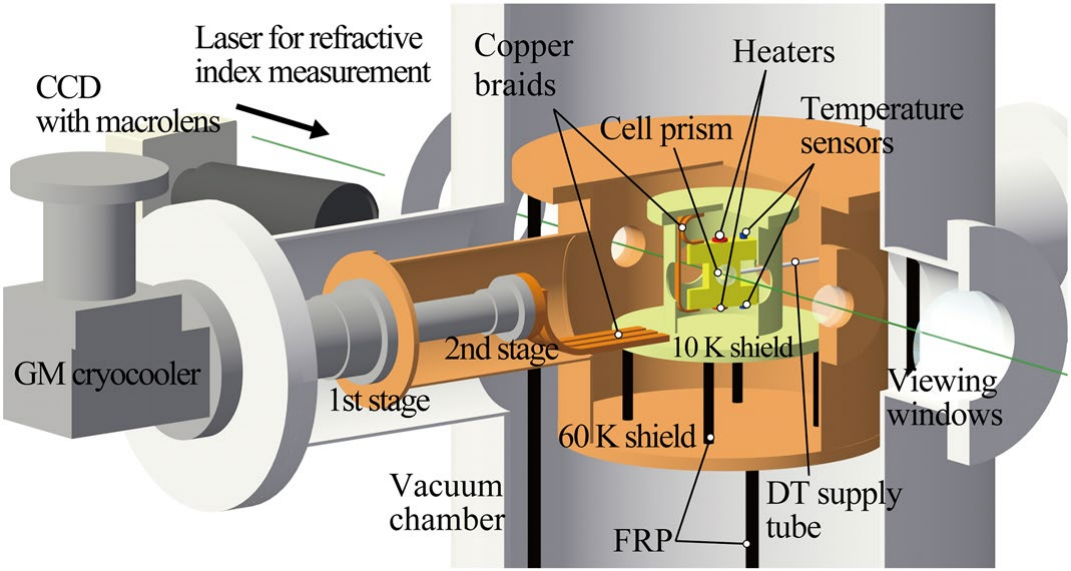
Cryogenic system for the measurement of refractive indexes.

The cryogenic system for refractive index measurements in liquid and solid phases consists of a prism cell, thermal shield, a vacuum chamber, a 10 K Gifford–McMahon (GM) cryocooler and a CCD camera as shown in Fig. 2. The cell prism is made of brass and glass for a body and optical components with a λ/10 optical flat, respectively. Indium is used to make the cell leak tight. To control the cell temperature, heaters and Cernox^®^ temperature sensors are attached on the top and the bottom of the cell, and Model 340 (Lake Shore Cryogenics, Inc., OH, USA) controlled the temperature with 1 mK precision. Thermal shields with optical windows are equipped at ~ 10 K and ~ 60 K and supported by Fiber Reinforced Plastic (FRP) rods to reduce heat influx from the room temperature. The GM cryocooler (RF-50A CR·GHe-10/80, Suzuki Shokan Co., Ltd., Tokyo, Japan) has the cooling ability of 4 W at 21 K. The first and second stages are connected with a ~ 60 K shield and the prism cell via a ~ 10 K shield, respectively. Copper braids as thermal links are applied to reduce vibration from the refrigerator^16^. The temperature of the prism cell can reach 12.7 K. A CCD camera (DS-5M, Nikon Corp., Tokyo, Japan) and macrolens (Micro Nikkor 200 mm, Nikon Corp., Tokyo, Japan) were used to observe the phase transition in the prism cell with backlighting techniques. At the cell temperature of ~ 20 K, the temperature difference between the top and the bottom of the cell was less than 10 mK without any control.

Before DT experiments, the refractive indexes of D_2_ (99.5%) and H_2_ (5 N) were measured in the liquid and solid phases. Instead of the gaseous DT supply system, a gaseous H_2_ cylinder was directly connected to the cryogenic system. After the top and the bottom temperature of the cell was controlled at 14.0 K just above the triple point, the prism cell was filled with H_2_ at the supply pressure of ~ 7.7 kPa. Then the temperature was lowered, and H_2_ was liquefied. The refractive index in the liquid phase was measured. The value was compared to a published data to calibrate the optical system for refractive index measurements. In the D_2_ measurements as a cold run, gaseous D_2_ was stored in the ZrNi bed in the DT supply system. The top and the bottom temperature of the prism cell was maintained at 18.8 K which is just above the triple point of D_2_, the ZrNi was heated more than 503 K to release D_2_ gas. The released D_2_ gas was loaded into the prism cell to liquefy the gaseous D_2_. The refractive index of liquid D_2_ was measured after the cell was filled with the liquid. When the top and the bottom temperature of the cell decreased to 18.69 K and 18.56 K, solidification was started. The refractive index of liquid and solid D_2_ were measured in the average temperature range of 16.80–18.78 K.

**Figure 3:**
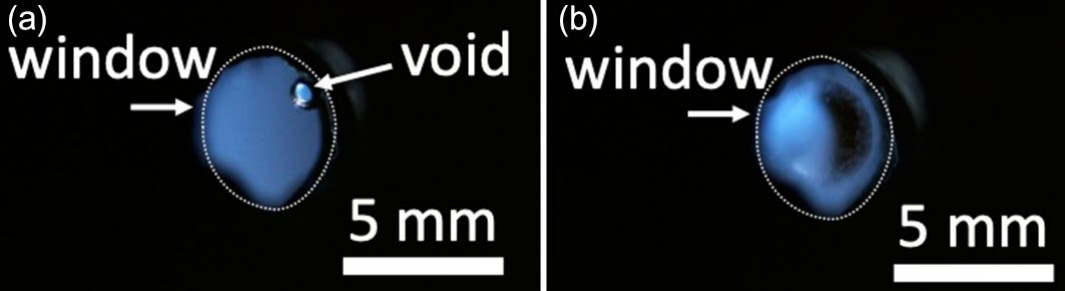
(**a**) DT liquefaction and (**b**) DT solidification in the cell.

For the DT experiments, after the top and bottom temperature of the cell was fined at 19.8 K, the triple point of DT, the ZrNi bed was heated at 673 K, and gaseous DT was released and supplied to the cell. Liquefaction of DT was observed (see Fig. 3a). The refractive index of liquid DT was measured after the cell was filled with liquid DT. Then, the temperature of the cell was gradually lowered. The top and bottom temperatures of the cell were controlled within a difference of ~ 0.12 K and the solidification was started at the bottom at 19.45 K. Because of volume reduction by the solidification, the vapor bubble remained at the top of cell (see Fig. 3b)^17^. The refractive index of solid DT was measured at optically flat point avoiding the vapor bubble. The average temperature of the cell was varied between 19.72 and 17.89 K to study the temperature dependence of the refractive index.

### Refractive index measurement

**Figure 4:**
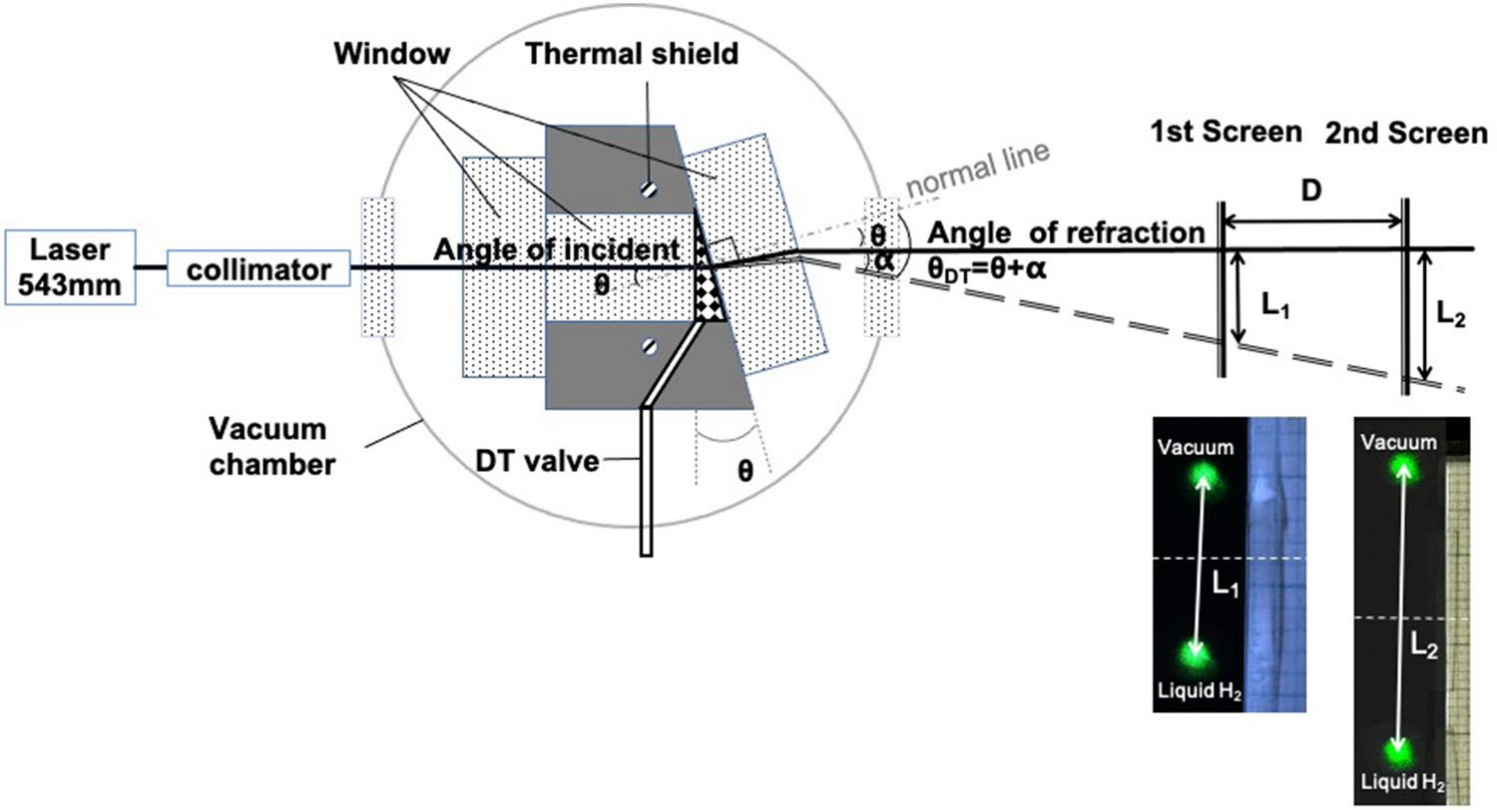
Schematic of optical measurements.

Figure 4 shows the schematic of the refractive index measurements and laser spots passing through a vacuum, the liquid or the solid. The D_2_ and DT liquid and solid are grown in the prism cell. The 543 nm incident laser was passed through a couple of quartz windows sealed with indium gaskets. The end of the cell is tilted at an angle of *θ*. The refracted angle for D_2_ or DT, *θ*_*x*_ (T) (*x* = D_2_ or DT) is calculated using

2 $${\theta }_{x}\left(T\right)=\mathit{arctan}\left[\frac{{L}_{2}-{L}_{1}}{D}\right]+\theta $$

where *L*_1_ and *L*_2_ are the shift between the laser spots through vacuum, D_2_ or DT on the 1st and 2nd screens, respectively. *D* is the distance between the 2 screens. The refractive index of D_2_ or DT, *n*_*x*_ (*x* = D_2_ or DT) is calculated using Snell’s law,

3 $${n}_{x}\cdot sin {\theta }_{x}\left(T\right)={n}_{0}\cdot sin\theta $$

The distance between the 1st and 2nd screens was 1035 mm. The laser spots on the screens were monitored with CCD cameras. The peak position of laser spot was analyzed using a gauss function fitting. The beam positions are located within the accuracy of ± 0.007 mm which was estimated from standard deviation. These uncertainties include the vibration noise of the experimental setup. The angle includes the uncertainty from the indium seal. This angle was calibrated using the liquid H_2_ refractive index of 1.120 at 15 K from Ref.^18^, was 9.43°.

## Results and discussion

**Figure 5:**
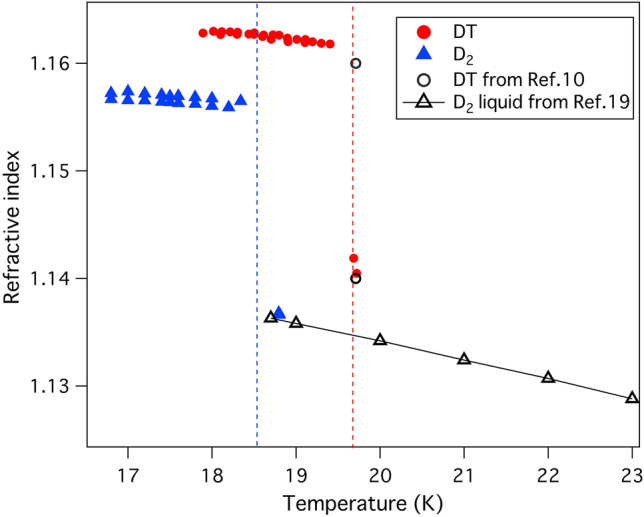
Refractive indexes of DT, D_2_ compared with that of liquid D_2_ from the empirical Eq. (3).

Figure 5 shows the refractive indexes of DT (54:45) and D_2_ as a function of the temperature. The refractive index of the solid DT at 19.404 K is 1.1618 ± 0.0002 and gradually increases to 1.1628 ± 0.0002 at 17.89 K. The refractive index of DT is summarized in Table 1. The uncertainty in refractive index was decided from the accuracy of beam positions estimated by gauss function fitting. The refractive indexes of liquid and solid D_2_ are 1.1367 ± 0.0005 at 18.80 K and 1.1564 ± 0.0008 at 18.34 K, respectively and increase gradually to 1.1567 ± 0.0005 at 16.80 K. The temperature dependence of the refractive index *dn/dT* was explained by Prod’homme using the relationship equation between thermal expansion coefficient and electrical polarizability. The *dn/dT* is given by differentiating Lorentz–Lorenz equation^19,20^

4 $$\frac{dn}{dT}=\frac{\left({n}^{2}-1\right)\left({n}^{2}+2\right)}{6n}(\Phi -\beta )$$

where *T* is the temperature, *β* is the volume thermal expansion coefficient and *Ф* is the polarizability coefficient defined as the temperature dependence of electronic polarizability. In case of hydrogen and their isotopologues, the refractive index increases with the density as described in Eq. (1). Thermal expansion has no wavelength dependence but the polarizability has dependent characteristics. At the edge of transparency or shorter wavelength range, the polarizability rises and *dn/dT* tends to become positive depending on the *Ф*. The solid hydrogen and its isotopes are typical molecular crystal. In general, molecular crystal has higher thermal expansion coefficient than covalent bond crystal, ionic crystal, and metal. In fact, the volume of solid hydrogen expands about 1% (linear expansion coefficient) from 0 to 14 K (triple points) while the general glass materials have 10^–5^–10^–6^ /K of linear expansion coefficient. This high thermal expansion coefficient leads to the negative *dn/dT*.

**Table 1:** The refractive index of liquid and solid DT.

Temperature (K)	n
19.72	1.1410 ± 0.0002
19.68	1.1421 ± 0.0002
19.40	1.1619 ± 0.0002
19.23	1.1620 ± 0.0002
19.19	1.1621 ± 0.0002
19.11	1.1623 ± 0.0002
19.10	1.1619 ± 0.0002
19.01	1.1624 ± 0.0002
18.90	1.1625 ± 0.0002
18.80	1.1627 ± 0.0002
18.72	1.1627 ± 0.0002
18.60	1.1627 ± 0.0002
18.50	1.1628 ± 0.0002
18.44	1.1627 ± 0.0002
18.30	1.1628 ± 0.0002
18.22	1.1629 ± 0.0002
18.12	1.1629 ± 0.0002
18.02	1.1629 ± 0.0002
17.89	1.1628 ± 0.0002

The refractive index of liquid D_2_ is in good agreement with that reported by Childs^21^. The calculated refractive indexes of solid D_2_ and liquid and solid DT (D:T = 1:1) at triple point are 1.155 ± 0.006, 1.140 ± 0.005, and 1.160 ± 0.006, respectively^22,23^. Measured refractive indexes are consistent with those from these references.

**Figure 6:**
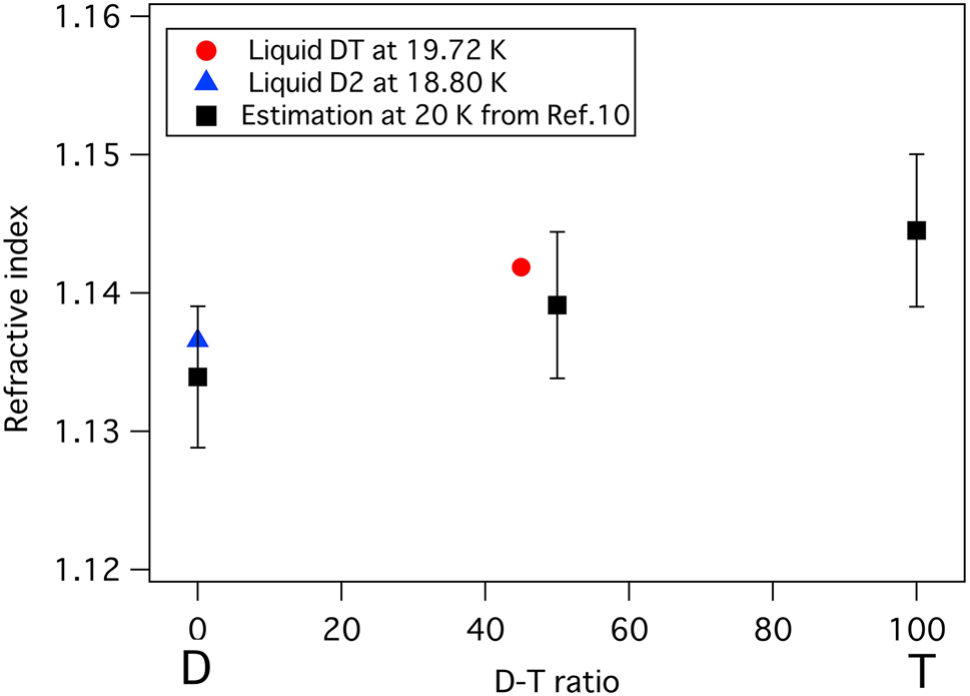
Comparison between the refractive indexes of liquid D_2_ at 18.80 K and liquid DT at 19.72 K at 543 nm and the refractive indexes at 20 K at 550 nm calculated from Eq. (3).

Figure 6 shows the refractive indexes of liquid D_2_ at 18.80 K and liquid DT at 19.72 K at 543 nm compared with the calculated refractive index at 20 K and 550 nm^10^. The calculated values with large uncertainties are slightly lower than our results.

## Conclusion

In conclusion, we have conducted the first refractive index measurements of liquid and solid DT in the range of 16.80–19.72 K. The refractive index of liquid DT is 1.1421 ± 0.0002 at 19.68 K and 543 nm. The refractive indexes of solid DT range from 1.1619 ± 0.0002 to 1.1628 ± 0.0002 in the temperature range of 19.40 K to 17.89 K. The refractive indexes of liquid and solid D_2_ are 1.1367 ± 0.0005 at 18.80 K and 1.1564 ± 0.0008 at 18.34 K, respectively and increase gradually to 1.1567 ± 0.0005 at 16.80 K.

## Data Availability

The datasets generated during and/or analyzed during the current study are available from the corresponding author on reasonable request.
